# Supplementary material for: A Chromosome-Length Assembly of the Hawaiian Monk Seal (Neomonachus schauinslandi): A History of “Genetic Purging” and Genomic Stability
Source: Genes (Basel). 2022 Jul 18;13(7):1270. doi: 10.3390/genes13071270 (PMC9323584; doi:10.3390/genes13071270)
Supplement: Supplementary file 1 [file genes-13-01270-s001.zip › genes-1789705-supplementary.pdf]

genes MDPI

genes-1789705

Supplementary Materials:

Table S1. Methods used in this study and the order in which they were employed.

Method	Platform	Input	Data type	Analysis tools
DISCOVAR	Illumina HS2500	500ng sheared DNA sheared to ~400 bp	2X250 PE reads	DISCOVAR *denovo* v52488
10X Genomics Chromium	Illumina HS2500	1.2ng HMW DNA (>40 kbp)	2X150 PE reads	Supernova v1.1 And V2.+
Optical genome mapping	Bionano Irys BspQ I	~10^7 cells ~160ng HMW DNA	>150 kbp molecules	BNG Assembler v5122
Oxford Nanopore	R9.1 Flow cell	1-2 ug HMW DNA ~9X read depth	N50 ~32 kbp	LINKS, Pilon
Hi-C, Arima	Illumina NS6500	~~~1 M reads	2X150 PE reads	3d-dna, Juicebox tools
Optical genome map 2	Saphyr DLE-1	~10^7 cells ~160ng HMW DNA	>150 kbp molecules	BNG Access
Synteny analysis			NCBI, DNAzoo assemblies	Minimap2, D-GENIES
Manual editing	Galaxy tools			

Table S2. Assembly sources for species comparisons

Common name	Species	Assembly	Source
Gray Seal	*Halichoerus grypus*	Halichoerus_grypus_HiC	DNAZoo
Harbor Seal	*Phoca vitulina*	GSC_HSeal_1.0_HiC	DNAZoo
Bearded Seal	*Erignathus barbatus*	Erignathus_barbatus_HiC	DNAZoo
N. Elephant Seal	*Mirounga angustirostris*	Mirounga_angustirostris_HiC	DNAZoo
**Hawaiian Monk Seal**	***Neomonachus schauinslandi***	**GCF_002201575.2_ASM220157v2**	**NCBI**
Antarctic Fur Seal	*Arctocephalus gazella*	GCA_900642305.1_arcGaz3	NCBI
Guadalupe Fur Seal	*Arctocephalus townsendi*		DNAZoo
California Sea Lion	*Zalophus californianus*	GCA_900631625.1_zalCal2.2	NCBI
Walrus	*Odobenus rosmarus divergens*	Oros_1.0_HiC	DNAZoo
Domestic cat	*Felis catus*	GCF_000181335.3_Felis_catus_9.0	NCBI
Domestic dog	*Canis familiaris*	GCF_000002285.3_CanFam3.1	NCBI

Table S3. Comparison of NCBI metrics for ASM220157v1 and ASM220157v2.

NCBI Global statistic	V1	V2
Total ungapped length	2,347,217,317	2,376,537,768
NCBI annotations		
Genes and Pseudogenes	24,730	27,736
Protein coding genes	18,856	19,838
Exons	204,392	212,563
Non-coding	1,173	3,122
Pseudogenes	4,701	4,734
mRNAs	28,368	34,516
tRNAs	446	531
lncRNA	824	780

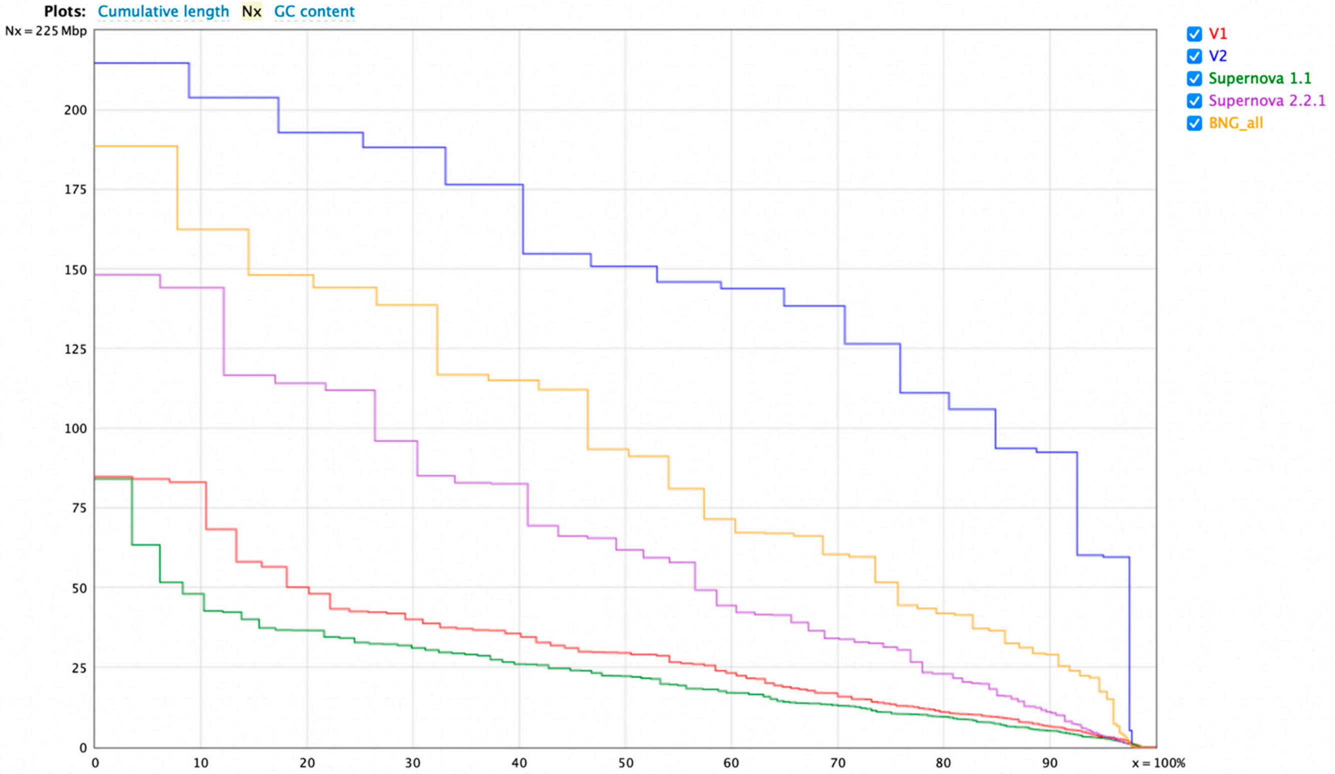

Figure S1. Improved assembly contiguity with additional methods. Linked reads with Supernova v1.1, N50=22.23 Mb; ASM220157v1 assembly, N50=29.52 Mb; Supernova 2.2.1 on original linked reads, N50=61.65 Mb; All methods with BNG maps, N50=93.39 Mb; ASM220157v2 assembly, 150.81 Mb.

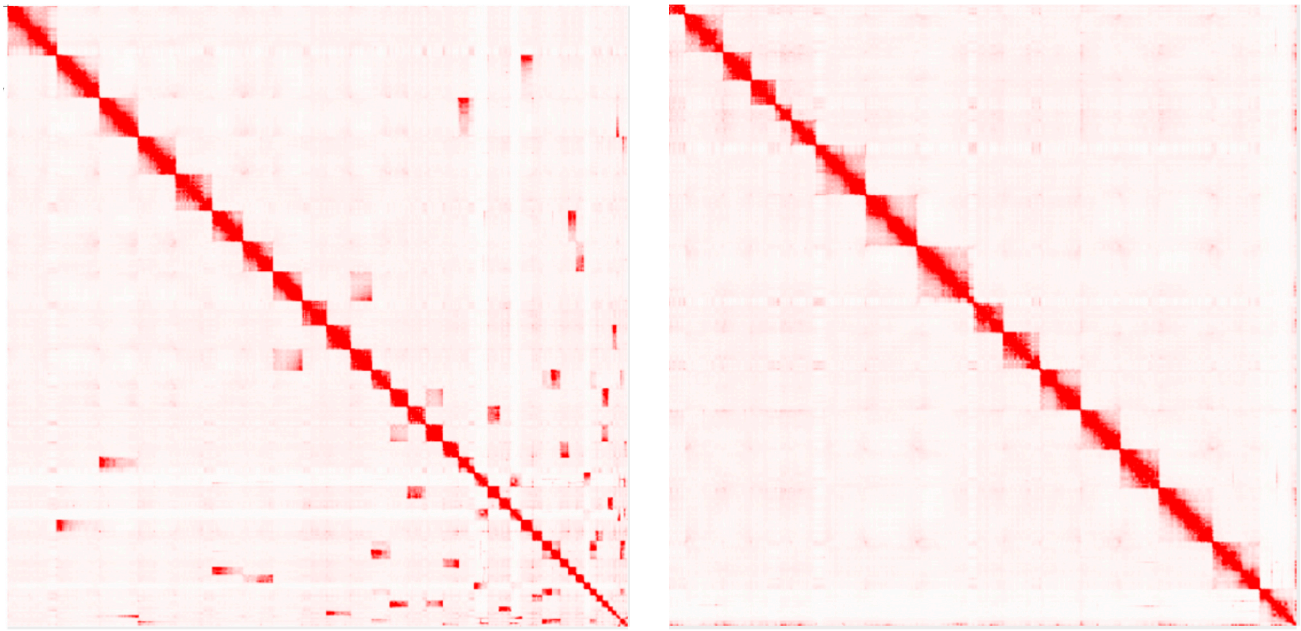

Figure S2. Hi-C chromosome-length assembly and Juicebox Assembly Tools polishing. Original plots and data are available at DNAzoo.org.

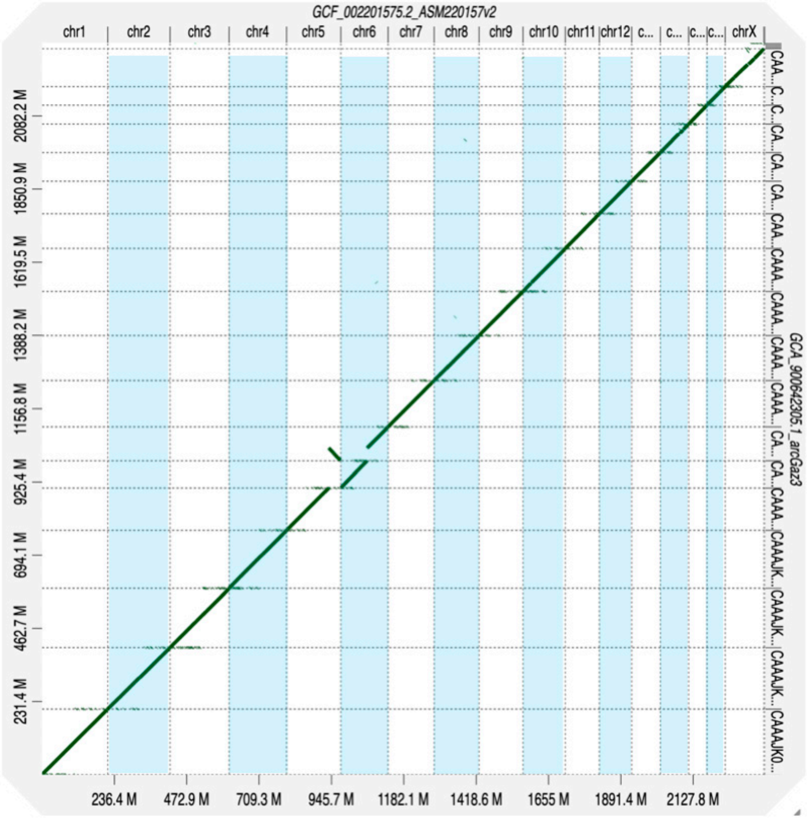

Guadalupe fur seal

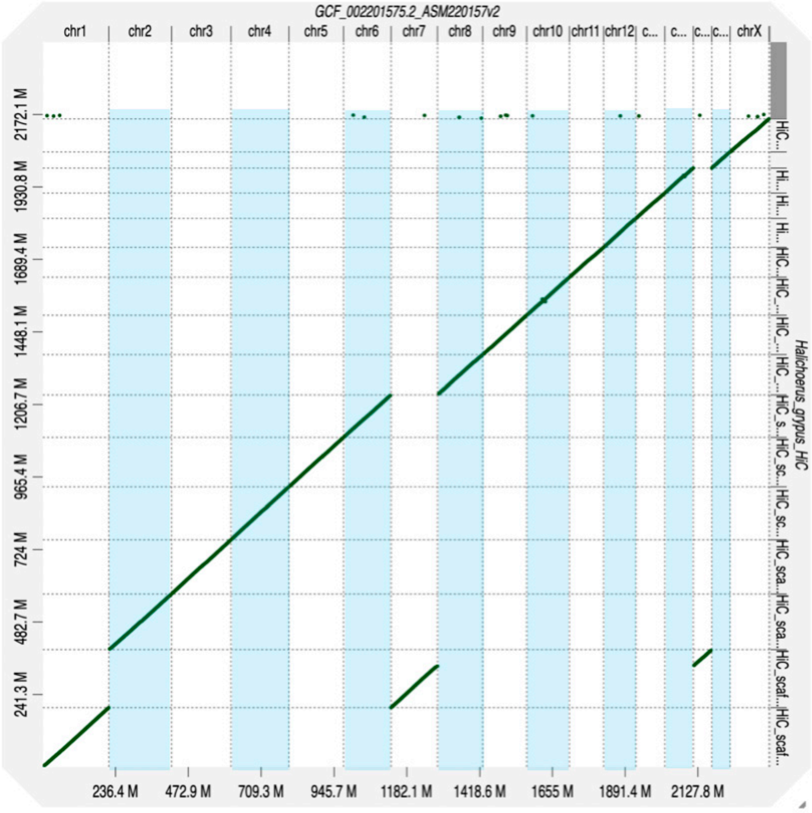
GCF_002201575.2_ASM220157v2
chr1
chr2
chr3
chr4
chr5
chr6
chr7
chr8
chr9
chr10
chr11
chr12
chrX
Halichoerus_grypus_HiC
2172.1 M
1930.8 M
1689.4 M
1448.1 M
1206.7 M
965.4 M
724 M
482.7 M
241.3 M
236.4 M
472.9 M
709.3 M
945.7 M
1182.1 M
1418.6 M
1655 M
1891.4 M
2127.8 M

Gray seal

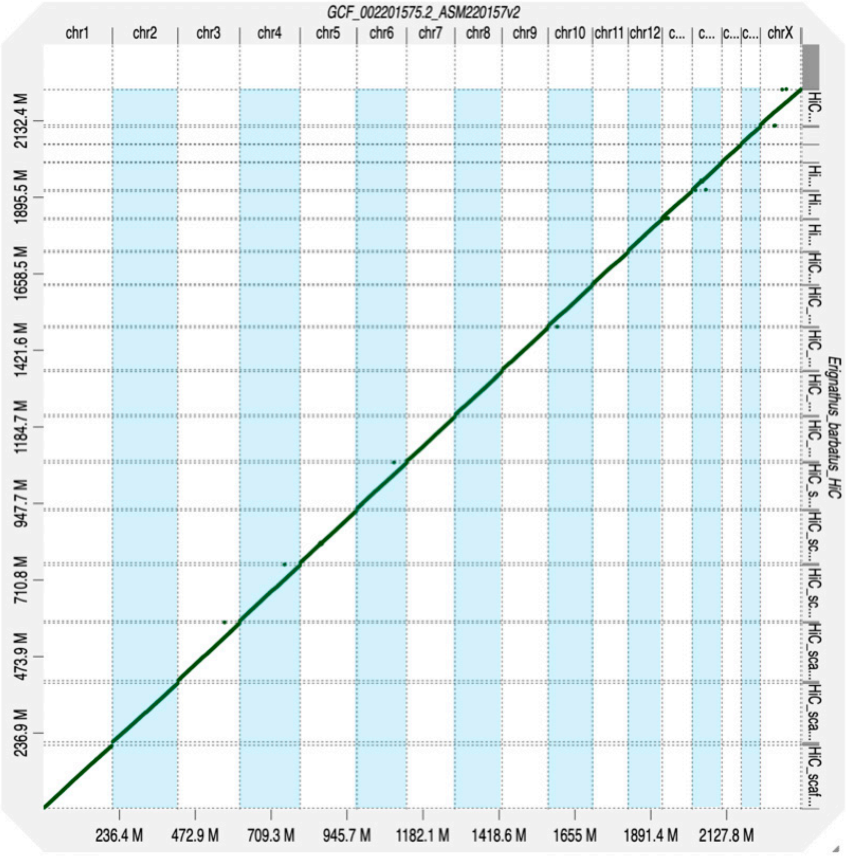

Bearded seal

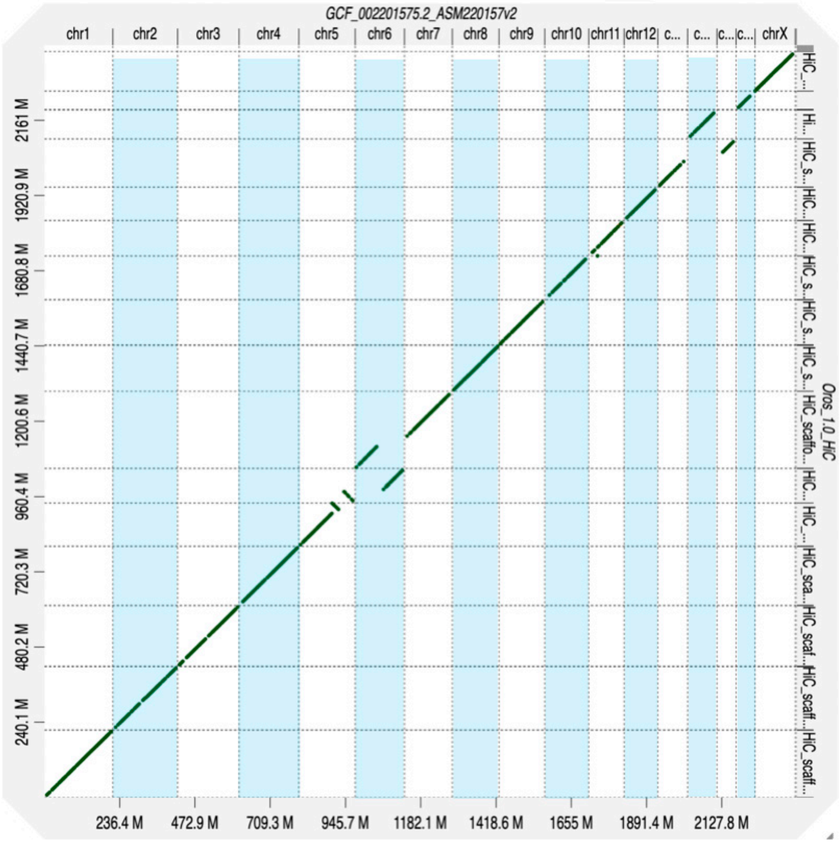
GCF_002201575.2_ASM220157v2
chr1
chr2
chr3
chr4
chr5
chr6
chr7
chr8
chr9
chr10
chr11
chr12
chrX
Oros_1.0_HiC
2161 M
1920.9 M
1680.8 M
1440.7 M
1200.6 M
960.4 M
720.3 M
480.2 M
240.1 M
236.4 M
472.9 M
709.3 M
945.7 M
1182.1 M
1418.6 M
1655 M
1891.4 M
2127.8 M

Walrus

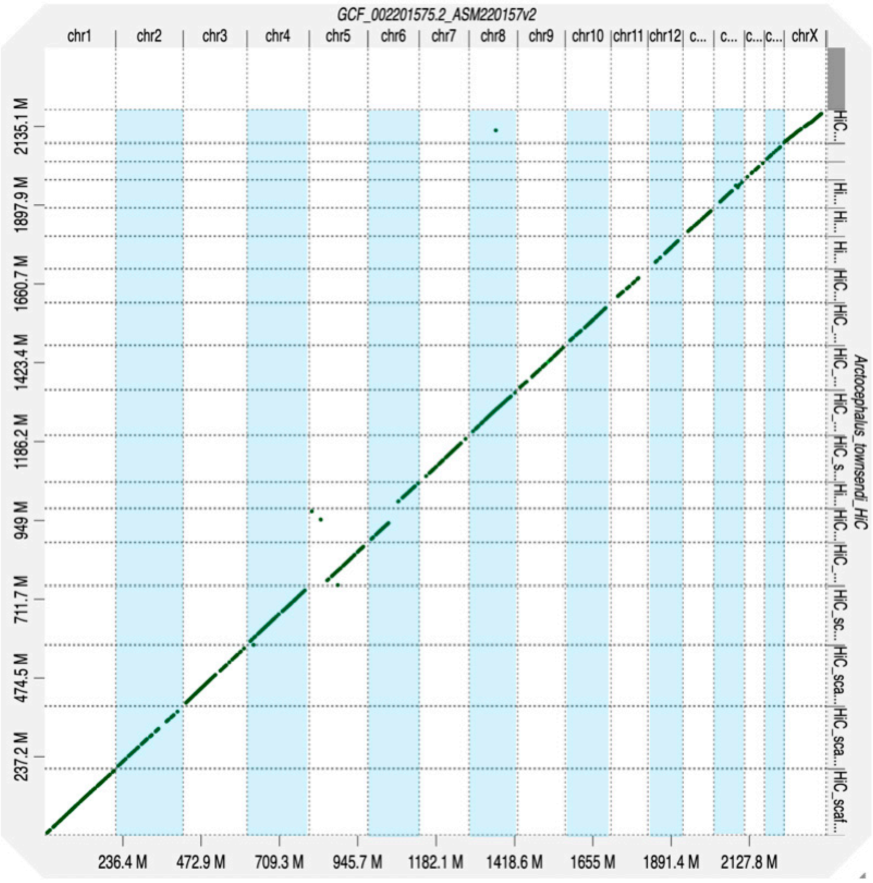

Antarctic fur seal

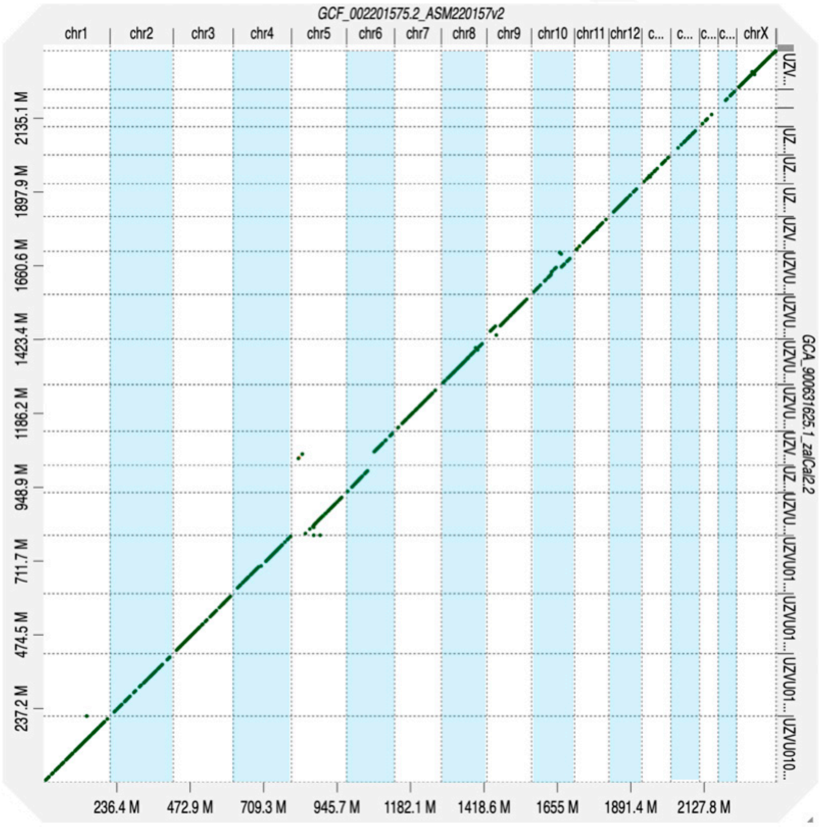

California sea lion

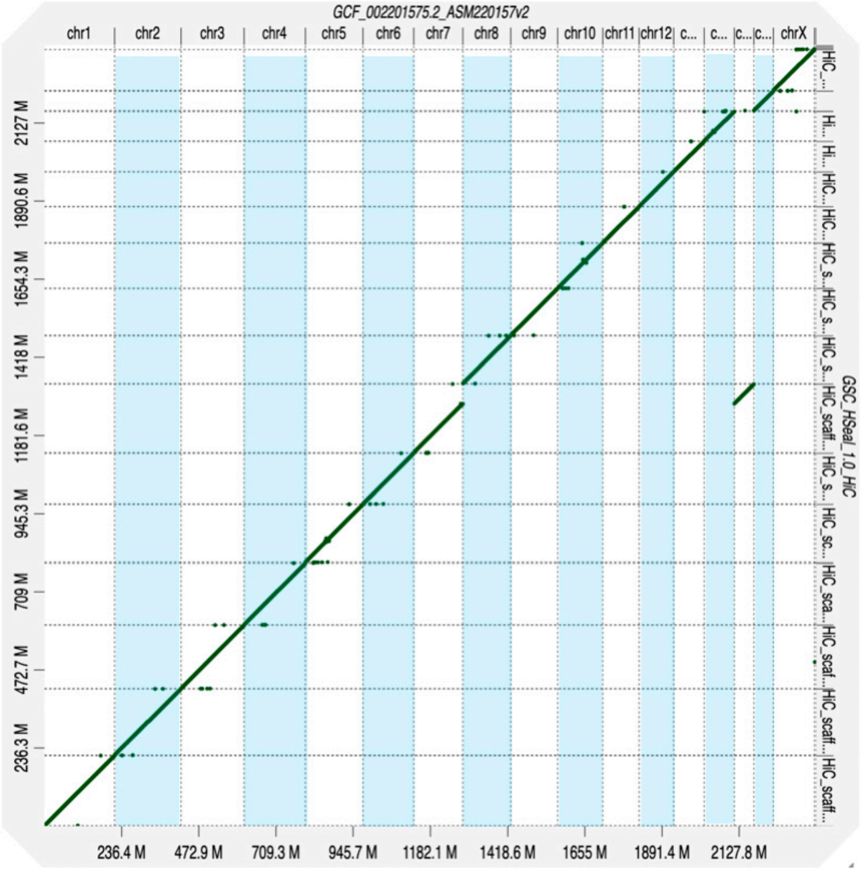

GCF_002201575.2_ASM220157v2
chr1
chr2
chr3
chr4
chr5
chr6
chr7
chr8
chr9
chr10
chr11
chr12
chrX
GSC_HSeal_1.0_HiC
236.4 M
472.9 M
709.3 M
945.7 M
1182.1 M
1418.6 M
1655 M
1891.4 M
2127.8 M
236.3 M
472.7 M
709 M
945.3 M
1181.6 M
1418 M
1654.3 M
1890.6 M
2127 M

Harbor Seal

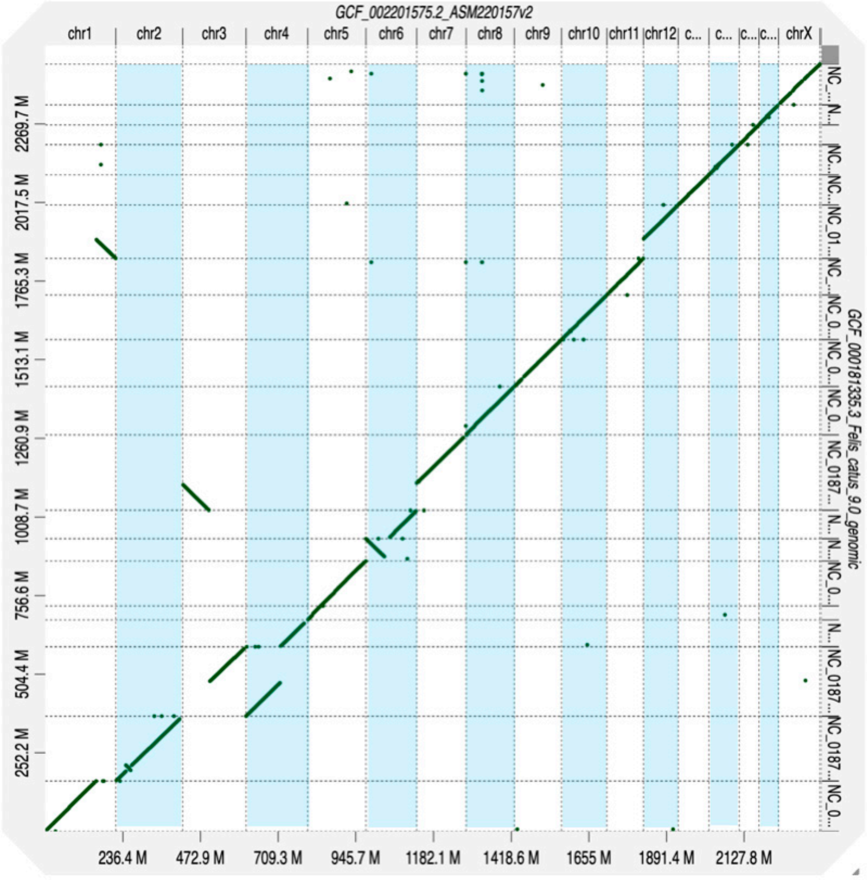
GCF_002201575.2_ASM220157v2
chr1
chr2
chr3
chr4
chr5
chr6
chr7
chr8
chr9
chr10
chr11
chr12
chrX
236.4 M
472.9 M
709.3 M
945.7 M
1182.1 M
1418.6 M
1655 M
1891.4 M
2127.8 M
252.2 M
504.4 M
756.6 M
1008.7 M
1260.9 M
1513.1 M
1765.3 M
2017.5 M
2269.7 M
GCF_000181335.3_Felis_catus_9.0_genomic

Cat

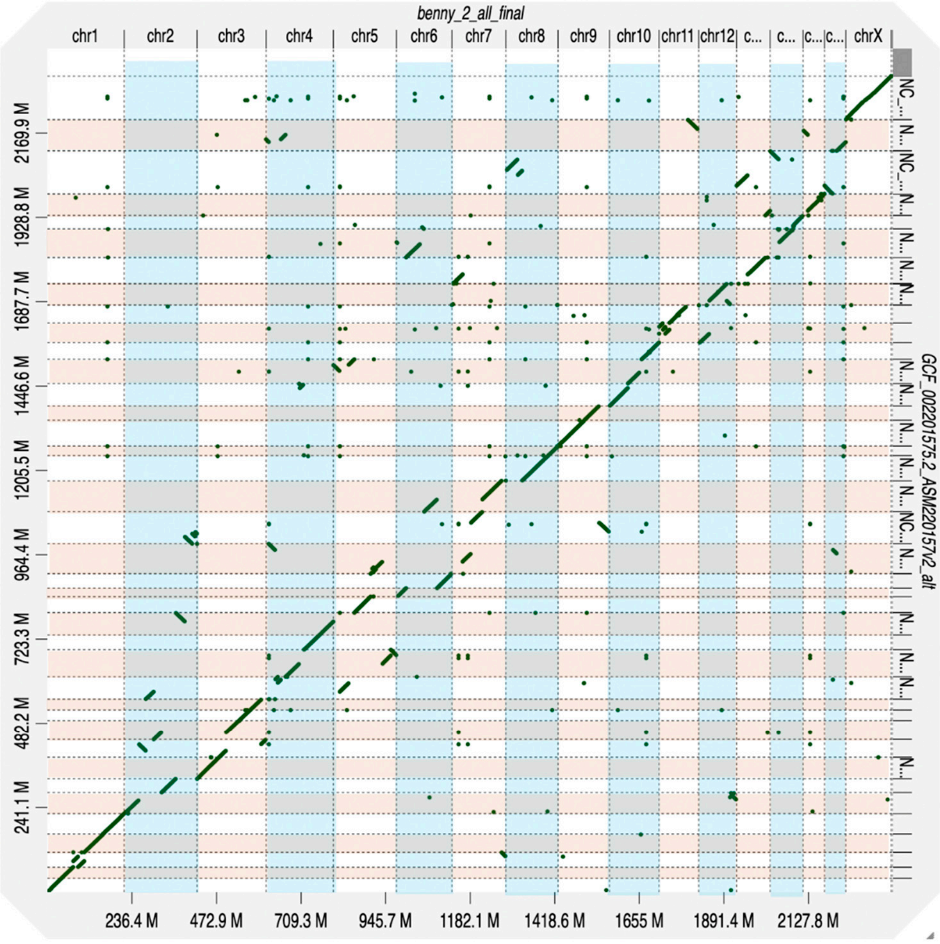

Dog

Alternate Hawaiian monk seal and dog chromosomes colored.

Figure S3. Enlarged DG plots of other species relative to HMS ASM220157v2. Selected chromosomes from other species were reverse-complemented before plotting.

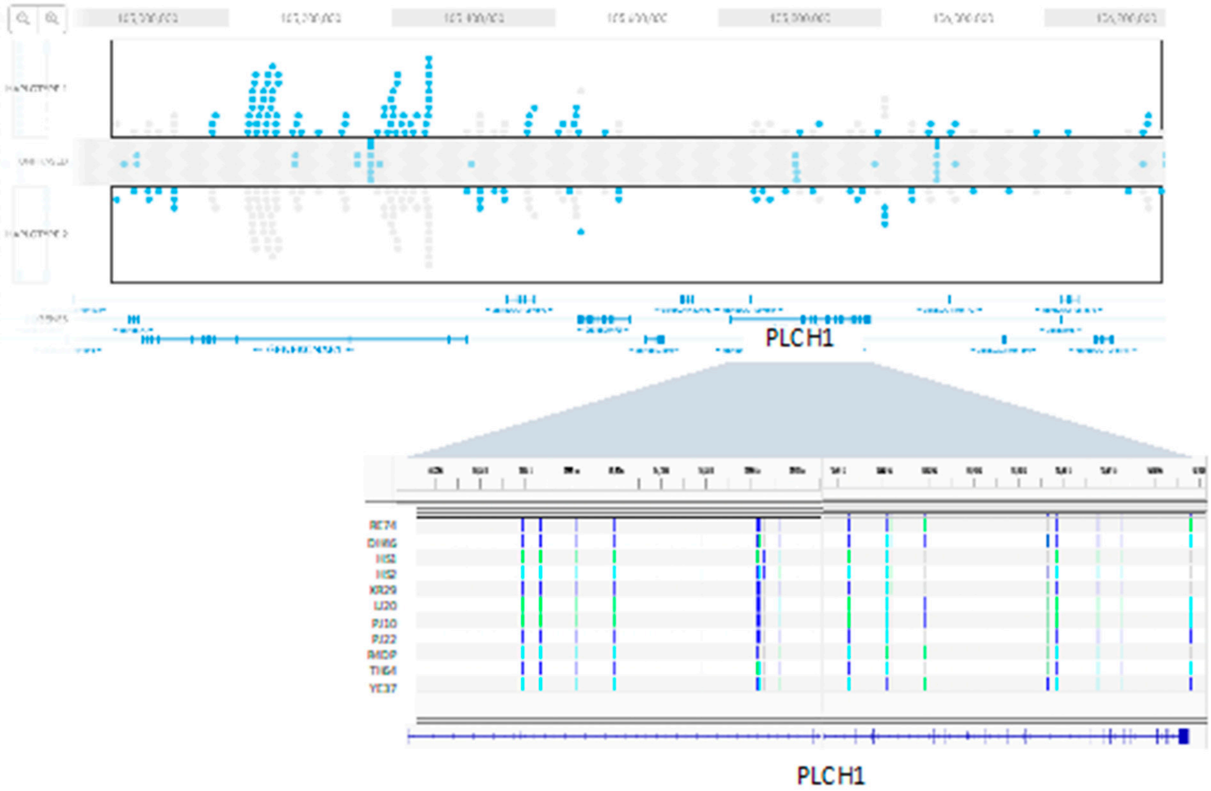

Figure S4. A representative 1.28Mb phase block in RE74 including KCNAB1 and PLCH1 shown in the Loupe viewer illustrating lack of heterogeneity among HMS. Alignment of variants in RE74 and 10 other seals within PLCH1. Solid dark blue bars are heterozygous differences from the RE74 reference, light blue are homozygous differences from reference and green bars are homozygous reference.
